# Association Between Oral Metformin Use and the Development of Age-Related Macular Degeneration in Diabetic Patients: A Systematic Review and Meta-Analysis

**DOI:** 10.1167/iovs.63.13.10

**Published:** 2022-12-09

**Authors:** Kai-Hsiang Liang, Chih-Hao Chen, Hou-Ren Tsai, Chun-Yu Chang, Tai-Li Chen, Wei-Cherng Hsu

**Affiliations:** 1 Department of Medical Education, Medical Administration Office, Taipei Tzu Chi Hospital, Buddhist Tzu Chi Medical Foundation, New Taipei City, Taiwan; 2 Department of Otolaryngology, Taipei Veterans General Hospital, Taipei City, Taiwan; 3 Department of Ophthalmology, Hualien Tzu Chi Hospital, Buddhist Tzu Chi Medical Foundation, Hualien, Taiwan; 4 Department of Anesthesiology, Taipei Tzu Chi Hospital, Buddhist Tzu Chi Medical Foundation, New Taipei City, Taiwan; 5 Department of Dermatology, Taipei Veterans General Hospital, Taipei City, Taiwan; 6 School of Medicine, Tzu Chi University, Hualien, Taiwan; 7 Department of Ophthalmology, Taipei Tzu Chi Hospital, Buddhist Tzu Chi Medical Foundation, New Taipei City, Taiwan

**Keywords:** age-related macular degeneration (AMD), metformin, meta-analysis

## Abstract

**Purpose:**

Metformin is a biguanide derivative that is commonly used for the treatment of diabetes mellitus (DM). It demonstrates antioxidative, anti-inflammatory, and antiangiogenic activity within the ocular tissue and thus may be implicated in the treatment of age-related macular degeneration (AMD). However, epidemiological studies have shown conflicting results.

**Methods:**

The Ovid MEDLINE/Embase, Cochrane Library, and Web of Science databases were systematically searched from inception through August 3, 2022. Studies reporting the association between metformin use and odds of AMD were enrolled. Adjusted odds ratios (ORs) of AMD were extracted and pooled with random-effects model meta-analysis. Subgroup analyses based on AMD subtypes, ethnicity, study design, sex, and confirmation of AMD diagnosis were conducted.

**Results:**

A total of 9 observational studies with 1,446,284 participants were included in the analysis. The meta-analysis showed that metformin use was associated with a significant reduction in the odds of AMD (pooled ORs = 0.81, 95% confidence interval [CI] = 0.70–0.93). Subgroup analyses revealed that metformin use was not significantly associated with dry or wet AMD. Black (pooled ORs = 0.61, 95% CI = 0.58–0.64) and Hispanic populations (pooled ORs = 0.85, 95% CI = 0.81–0.89) demonstrated significantly lower odds of AMD.

**Conclusions:**

This systematic review and meta-analysis found that patients with DM with metformin usage were at lower odds of developing AMD. Future prospective clinical trials are needed to confirm this association.

Age-related macular degeneration (AMD) was the fourth most common cause of blindness in those aged 50 years and older in 2020 globally, with 1.8 million people affected.^1^ Patients plagued by AMD are projected to rise from 196 million in 2020 to 288 million in 2040.^2^ AMD can be divided into wet (neovascular) and dry (non-neovascular) subtypes. Dry AMD accounts for approximately 90% of total AMD cases.^3^ However, effective treatment is still lacking.

Metformin, a biguanide derivative, is the preferred first-line treatment in type 2 diabetes mellitus (DM). Apart from its antiglycemic effect, it was shown to possess antiaging properties.^4^ In retinal tissues, metformin demonstrated antioxidative,^5^ anti-inflammatory,^6^ anti-angiogenic,^7^ and pro-autophagic effects^8^ mediated through activation of 5' adenosine monophosphate-activated protein kinase (AMPK). As the pathogenesis of AMD involves oxidative stress,^9^ inflammation,^10^ neovascularization, and autophagic dysfunction,^11^ metformin may have potential in preventing or treating this sight-threatening disease. However, the results have been inconsistent in various studies.^12^^–^^20^

A recent meta-analysis investigated the possible association between metformin and the development of AMD.^21^ Nevertheless, it failed to demonstrate statistical significance in decreasing the odds of AMD with metformin usage. The limited number of included studies also precluded subgroup analysis on outcomes, including AMD subtypes and further stratification based on ethnicity. Since then, four additional cohort studies, including one prospective study, have been performed, again presenting conflicting results. Therefore, a systematic review and meta-analysis that incorporates all currently available evidence on this topic is warranted to establish the conducive effect of metformin usage on AMD.

## Methods

The systematic review and meta-analysis were undertaken in accordance with the Preferred Reporting Items for Systematic Reviews and Meta-Analyses (PRISMA) 2020 guidelines. The title and protocol were registered prospectively on the International Prospective Register of Systematic Reviews database (CRD42022353750).

### Search Strategy

We systematically performed eligibility screening with the Ovid MEDLINE/Embase, Cochrane Library, and Web of Science databases from inception through August 3, 2022, aiming for studies that reported the relationship between oral metformin use and the odds of AMD. Detailed search strategies with syntax specific for each database are available in Supplementary Table S2. References from the included studies and relevant reviews underwent further screening to account for potential additional studies. No language restrictions were applied during the search process.

### Eligibility Criteria

Studies were included if the following criteria were met: (1) observational studies (including cross-sectional, cohort, or case‒control designs); (2) studies enrolling patients diagnosed with DM; (3) intervention group actively prescribed oral metformin; (4) comparator group consisted of metformin nonusers at the time of recruitment; and (5) articles reporting the association between oral metformin use and AMD. Studies were excluded if the diagnosis of AMD preceded DM or if other macular diseases were present. Studies without control groups, animal studies, case reports or series, reviews, and conference abstracts with inadequate provision of data were also excluded.

### Data Extraction and Risk of Bias Assessment

Two reviewers (authors K.L. and H.T.) separately screened the title and abstract of the articles retrieved from the preliminary search. Full texts from potentially eligible studies were downloaded for screening to identify the final included studies. Disparities during the decision process were resolved by discussion between the two reviewers. The two reviewers (authors K.L. and H.T.) then independently extracted the following data from the included studies: first author, date of publication, country, study period, database utilized, mean age of the cohort, follow-up duration, participants exposed/nonexposed to metformin, and the method for ascertainment of AMD diagnosis. Discrepancies were first discussed between the two reviewers. The third author (C.C.) was consulted if disagreements could not be resolved. The risk of bias of the individual observational studies was independently assessed by the two reviewers (authors K.L. and H.T.) with the Risk of Bias in Non-randomized Studies of Exposure (ROBINS-E) assessment tool.^22^ Consensus was reached between the two reviewers should differences in grading results arise.

### Data Synthesis and Statistical Analysis

For studies providing incomplete data for meta-analysis, the corresponding authors of each study were contacted for additional information. We performed meta-analyses with RevMan version 5.4.1 software version (The Cochrane Collaboration). For dichotomous outcomes, pooled estimates were reported as odds ratios (ORs) with their respective 95% confidence intervals (CIs). The OR with the most covariates adjusted within each study was selected for analysis. The adjusted ORs of the sole use of metformin were used for synthesis if provided. A *P* value less than 0.05 was considered statistically significant. Due to the expectation of differences in treatment effect, the DerSimonian–Laird random-effects model was used to calculate the effect estimates.

Between-study heterogeneity was assessed with I^2^ statistics. An I^2^ value between 50 and 75% was defined as moderate heterogeneity, and >75% was considered to be of substantial heterogeneity.^23^ To account for possible heterogeneity, subgroup analyses were conducted according to predetermined categories, including subtypes of AMD, ethnicity, study designs, sex, and the ascertainment of AMD diagnosis. Sensitivity analysis was performed by removing one study at a time from the analysis (leave-one-out analysis). Studies at high risk of bias were also excluded in the sensitivity analysis. Publication bias will be evaluated with the asymmetry of funnel plots if more than 10 studies are enrolled in the final analysis.

Additionally, we conducted trial sequential analysis (TSA) with TSA software version 0.9.5.10 Beta to determine whether the required information size (RIS) was met to confirm the treatment benefits.^24^ We set the alpha value at 5% with a power of 80%. The relative risk reduction was set at 20%. If the cumulative z-curve crosses the trial sequential monitoring boundaries without falling into the inner wedge futility boundaries, the treatment effect will be regarded as significant.^25^

## Results

### Study Selection

**Figure 1.**
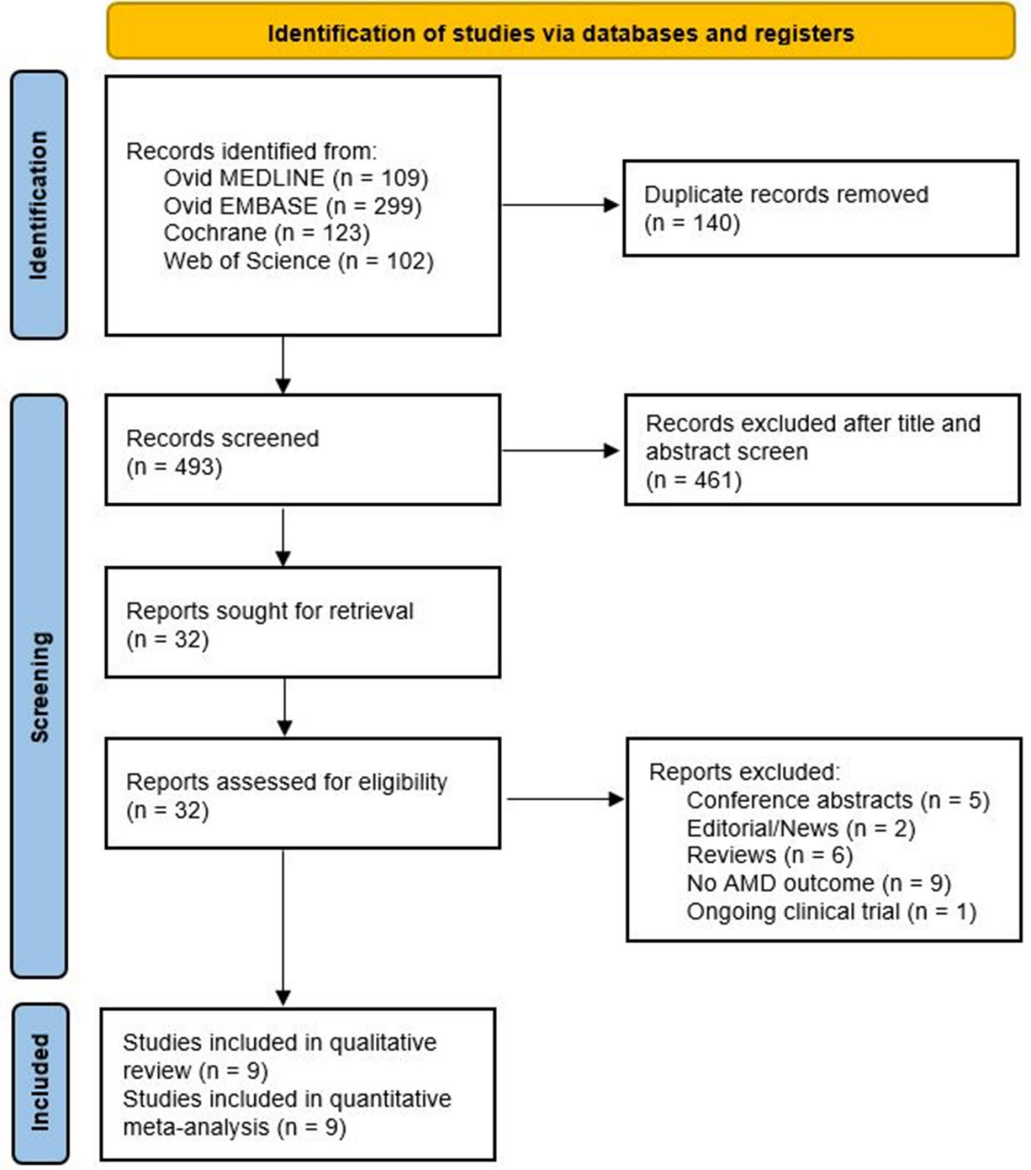
**PRISMA flow diagram.** AMD, age-related macular degeneration.

The PRISMA flowchart for the database search results is presented in Figure 1. After the removal of duplicate records, a total of 493 studies underwent title and abstract screening. The full texts of 32 studies were retrieved for assessment of eligibility, with 9 studies remaining in the final quantitative meta-analysis.^12^^–^^20^

### Characteristics of Included Studies

Baseline characteristics and pertinent outcomes are presented in Table 1. A total of 9 studies with 1,446,284 participants were enrolled in the study. The included studies consisted of one cross-sectional,^19^ three case‒control,^12^^,^^13^^,^^18^ and five cohort studies.^14^^–^^17^^,^^20^ Eight studies were retrospective in design,^12^^–^^19^ and one was conducted prospectively.^20^ Four studies were performed in the United States,^12^^,^^13^^,^^15^^,^^19^ three in Asia,^14^^,^^17^^,^^18^ and the other two in Europe.^16^^,^^20^ Seven studies obtained a diagnosis of DM with International Classification of Diseases (ICD) codes,^12^^–^^15^^,^^17^^–^^19^ one with database read codes,^16^ and one with medical records.^20^ Four studies exclusively enrolled patients with type 2 DM,^14^^,^^16^^,^^17^^,^^20^ whereas 5 studies also recruited patients with type 1 DM.^12^^,^^13^^,^^15^^,^^18^^,^^19^ Seven studies investigated the outcome of incident AMD,^12^^–^^19^ whereas one study aimed for both incident and prevalent AMD.^20^ The adjusted covariates applied in each study are illustrated in Supplementary Table S3.

**Table 1.** Characteristics of Included Studies

Studies	Country	Study Period	Database	Age, y, Mean (SD)	No. of Participants (Exposed/Nonexposed)	Follow-Up Periods, y	Definition of Metformin Use	Definition of AMD Diagnosis	OR (95% CI) for AMD
Brown et al. (2019)^13^	United States	2011–2017	University of Florida Integrated Data Repository	75.5 (9.0)^*^	660/4287	NA	RxNorm IDs	ICD-9 codes	0.70 (0.49, 0.98)
Chen et al. (2019)^14^	Taiwan	2001–2013	Taiwan NHIRD	56.1 (12.6)	45524/22681	6.7 (3.7)	National Drug Code	ICD-9 codes	0.57 (0.52, 0.63)
Lee et al. (2019)^18^	South Korea	2012–2015	NHIS-NEC	66.4 (5.0)^*^	1173/24435^*^	NA	Prescription history	ICD-10 codes	1.15 (0.91, 1.45)^*^
Stewart et al. (2020)^19^	United States	2012–2019	UCSF EMR	60+	1807/1313	NA	Medical record	ICD-9 & ICD-10 codes	0.70 (0.55, 0.88)
Blitzer et al. (2021)^12^	United States	2008–2017	IBM MarketScan	74.9 (10.3)^*^	76968/83791	NA	National Drug Code	ICD-9 & ICD-10 codes	0.95 (0.93, 0.97)
Eton et al. (2022)^15^	United States	2002–2016	Clinformatics Data Mart	67.5 (8.9)	166114/841111	NA	NA	ICD-9 & ICD-10 codes	1.08 (1.04, 1.12)
Gokhale et al. (2022)^16^	United Kingdom	1995–2019	IMRD-UK	62.8 (11.6)	154016/19673	5.7 (4.1)	Medical record	Database read codes	1.01 (0.91, 1.12)
Jiang et al. (2022)^17^	China	2015–2020	EMR	67 (1.8)	209/115	NA	Daily average dose >250mg	ICD-10 codes	0.24 (0.13, 0.42)
Vergroesen et al. (2022)^20^	Netherlands	1990–2014	Rotterdam study	65.1 (9.8)^*^	152/2254	8.2 (3.4)	Anatomical therapeutic chemical code	Rotterdam classification	0.54 (0.29, 1.00)

* Participants not limited to diabetic patients.

AMD, age-related macular degeneration; CI, confidence interval; EMR, electronic medical record; IBM, International Business Machines Corporation; ICD, International Classification of Diseases; ID, identifier; IMRD, IQVIA (IMS Health, Quintiles, VIA) Medical Research Data; NA, not available; NHIRD, National Health Insurance Research Database; NHIS-NEC, National Health Insurance Service-National Elderly Cohort; OR, odds ratio; SD, standard deviation; UCSF, University of California, San Francisco; y, year.

### Risk of Bias Assessment

Supplementary Figure S1 summarizes the risk of bias assessment for each study. Four studies were rated with a high risk of bias in the domain of confounding,^13^^–^^15^^,^^18^ which was due to the absence of adjustment for smoking status, a known risk factor for AMD.^26^ The study by Blitzer et al. was judged to have some concerns for bias in the same domain, as ethnicity was not included in the adjusted covariates.^12^

### Association of Oral Metformin Use With AMD

**Figure 2.**
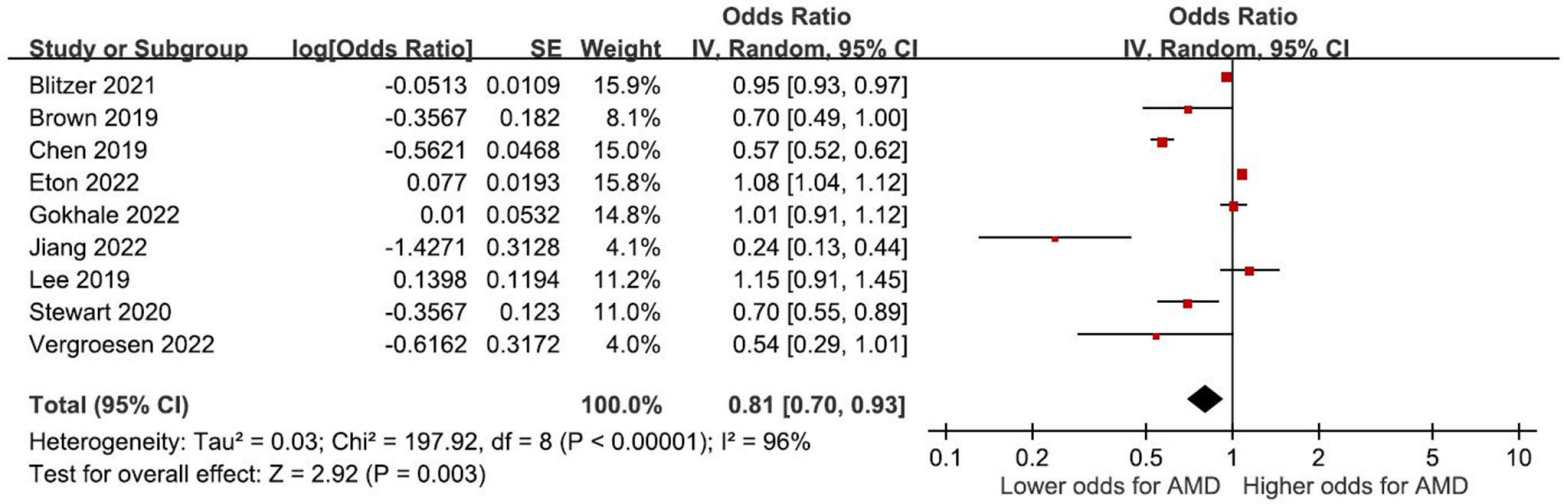
**Association of oral metformin use with AMD.** AMD, age-related macular degeneration; CI, confidence interval; IV, inverse variance; SE, standard error.

**Figure 3.**
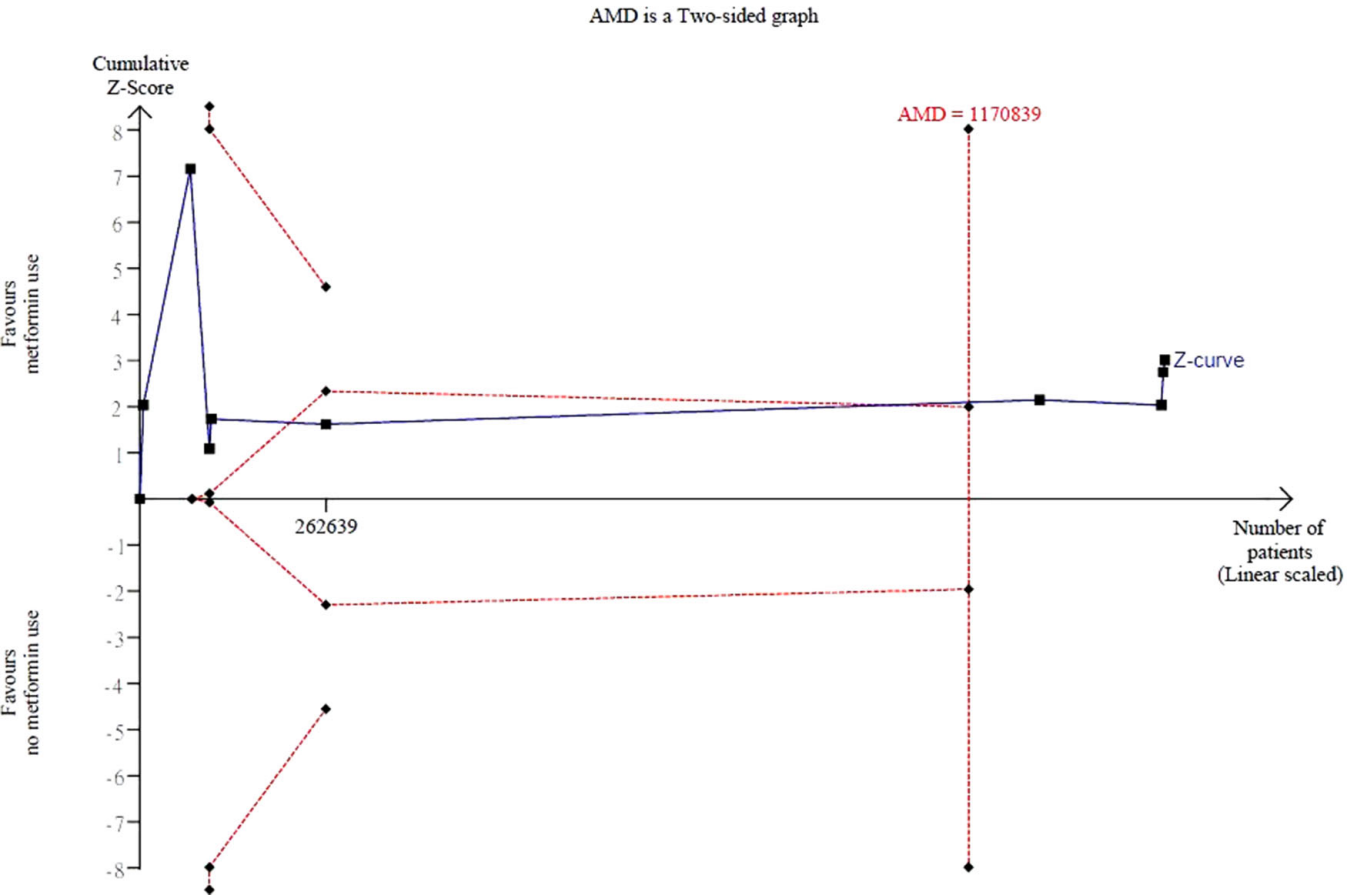
**Trial sequential analysis (TSA).** AMD, age-related macular degeneration. Trial sequential analysis of 9 observational studies included in the meta-analysis showed that the required information size of 1,170,839 was exceeded.

A total of 9 observational studies with 1,446,284 patients reported multivariable adjusted ORs. Meta-analysis revealed a significant decrease in the odds of AMD for oral metformin use (pooled ORs = 0.81, 95% CI = 0.70–0.93, *P* < 0.01, I^2^ = 96%; Fig. 2). TSA demonstrated that the RIS (*n* = 1,170,839) was exceeded. In addition, the cumulative z-curve crossed the trial sequential monitoring boundary, indicating that the decrease in the odds of AMD observed from conventional meta-analysis was further confirmed (Fig. 3).

Table 2 presents the results for subgroup analysis according to AMD subtypes, ethnicity, study design, sex, and confirmation of AMD diagnosis. The stratified analysis showed no significant association between metformin use and either dry (pooled ORs = 0.98, 95% CI = 0.62–1.57, *P* = 0.94, I^2^ = 92% Supplementary Fig. S2) or wet AMD (pooled ORs = 1.03, 95% CI = 0.80–1.22, *P* = 0.82; see Supplementary Fig. S2).

**Table 2.** Association of Oral Metformin Use With Age-Related Macular Degeneration

		Age-Related Macular Degeneration			Subgroup Difference	
Subgroups	No. of Studies	Pooled OR (95% CI)	*P* Value	I^2^ (%)	*P* Value	I^2^ (%)
AMD subtypes					0.86	0
Dry AMD	3	0.98 (0.62 to 1.57)	0.94	92		
Wet AMD	1	1.03 (0.80 to 1.33)	0.82	NA		
Ethnicity					<0.01^**^	96.6
Asian	4	0.71 (0.40 to 1.27)	0.25	99		
Black	2	0.61 (0.58 to 0.64)	<0.01^**^	0		
Caucasian	2	0.91 (0.36 to 2.33)	0.85	88		
Hispanic	1	0.85 (0.81 to 0.89)	<0.01^**^	NA		
Study design					0.18	43.8
Cohort studies	5	0.67 (0.47 to 0.95)	0.03^*^	98		
Others	4	0.88 (0.73 to 1.07)	0.20	74		
Sex					0.09	65.1
Male	4	0.91 (0.79 to 1.04)	0.16	92		
Female	2	1.06 (0.94 to 1.21)	0.34	74		
Ascertainment of AMD					0.25	24
ICD codes	7	0.79 (0.67 to 0.93)	<0.01^**^	97		
Clinical diagnosis	1	0.54 (0.29 to 1.01)	0.05	NA		

According to subgroups.

AMD, age-related macular degeneration; NA, not applicable.

* *P* < 0.05.

** *P* < 0.01.

Regarding ethnicity, the analysis demonstrated a significant decrease in the odds of AMD in Black (pooled ORs = 0.61, 95% CI = 0.58–0.64, *P* < 0.01, I^2^ = 0%; Supplementary Fig. S3) and Hispanic populations (pooled ORs = 0.85, 95% CI = 0.81–0.89, *P* < 0.01; see Supplementary Fig. S3), whereas no significant association was found in Caucasian (pooled ORs = 0.91, 95% CI = 0.36–2.33, *P* = 0.85, I^2^ = 88%; see Supplementary Fig. S3) or Asian populations (pooled ORs = 0.71, 95% CI = 0.40–1.27, *P* = 0.25, I^2^ = 99%; see Supplementary Fig. S3). Subgroup analysis based on study design exhibited a significant association between metformin use and AMD in cohort studies (pooled ORs = 0.67, 95% CI = 0.47–0.95, *P* = 0.03; I^2^ = 98%; Supplementary Fig. S4), whereas the association was nonsignificant in non-cohort studies (pooled ORs = 0.88, 95% CI = 0.73–1.07, *P* = 0.20; I^2^ = 74%; see Supplementary Fig. S4).

### Sensitivity Analysis

The results obtained by excluding studies with a high risk of bias yielded results similar to those of the primary analysis. On the other hand, the leave-one-out analysis overall remained statistically significant, with the exception of the removal of the study by Chen et al., which showed nonsignificant results (Supplementary Table S4).

## Discussion

This meta-analysis of 9 observational studies with 1,446,284 participants concluded that oral metformin use was associated with 0.81-fold decreased odds of AMD. However, metformin use did not decrease the odds of dry or wet AMD. Black and Hispanic populations seemed to benefit most from metformin use.

An earlier meta-analysis combined the results from five retrospective studies, showing a nonsignificant association between metformin use and the risk of AMD (OR = 0.80, 95% CI = 0.54–1.05). Subgroup analysis was not performed due to the few studies enrolled.^21^ Compared to the previous review, 4 studies (including one prospective study) with 1,183,645 patients were added to the analysis. Furthermore, the additional studies included data from European countries, which improved the generalizability of our results. Subgroup analyses based on ethnicity, sex, and study design could partially account for the substantial heterogeneity encountered in the primary analysis. Additionally, the marked heterogeneity of the pooled estimates may stem from the disparities among the adjusted covariates in each study (see Supplementary Table S3), which was commonplace in meta-analyses of observational studies.^27^

Metformin was shown to exert its therapeutic effect against AMD via diverse mechanisms. First, metformin activates AMPK, which leads to subsequent liver X receptor (LXR) activation and adenosine triphosphate-binding cassette (ABC) transporters ABCA1/G1 expression, reducing intracellular cholesterol accumulation and therefore drusen formation.^28^^,^^29^ Second, oxidized low-density lipoprotein derived from drusen induces inflammation and oxidative stress.^30^^–^^33^ In acute retinal pigment epithelial-19 (ARPE-19) cells exposed to oxidative stress induced by glyoxal, metformin was demonstrated to reverse the oxidative damage via AMPK activation.^5^ Third, metformin was found to possess anti-angiogenic activity in human retinal vascular endothelial cells,^7^ which might implicate a possible role in neovascular AMD. Finally, retinal pigment epithelium (RPE) cells are pivotal in maintaining normal photoreceptor function by phagocytosing the shed outer segments of photoreceptors.^34^ Senescent RPE cells, therefore, resulted in dysfunctional photoreceptors. Mechanistic target of rapamycin (mTOR) inhibition by AMPK induces autophagy in RPE cells, which serves to maintain effective self-clearance.^34^^–^^36^ Thus, metformin might have the potential to prevent RPE cell degeneration and AMD mediated by AMPK activation.^37^

Subgroup analysis based on subtypes of AMD did not show a significant association between oral metformin use and non-neovascular or neovascular forms of AMD, which was contrary to the proposed mechanisms delineated in the previous paragraph. This discrepancy may result from the heterogeneous population enrolled in Lee et al.’s study,^18^ where patients with cardiovascular disease were also included. Another possible explanation was that other antidiabetic medications may be used concomitantly with metformin, such as sulfonylurea and insulin, which might be a potential source of confounding. In the subgroup analysis for dry AMD, two studies did not specify whether other antidiabetics were prescribed.^15^^,^^19^ Previous studies found that ABCA1 and scavenger receptor BI (SR-BI) were expressed in cultured human RPE cells,^38^^,^^39^ both of which are involved in reverse cholesterol transport and lipid efflux.^40^^,^^41^ Glyburide (a sulfonylurea drug) inhibits ABCA1 and SR-BI activity,^42^ which in turn impedes efflux of lipids derived from phagocytosed photoreceptor outer segments from cultured RPE cells.^39^ This observed effect of sulfonylurea might play a role in the pathogenesis of dry AMD, thus resulting in a nonsignificant treatment benefit of metformin.

Our results showed that metformin use conferred a more significant treatment benefit in the Black population. Various population-based cohort studies have demonstrated that the risk of AMD was lower in Black populations than in White populations,^43^^,^^44^ which was attributed to the protective effect of ocular pigmentation and the higher frequencies of protective allele expression.^45^^,^^46^ Our observation may result from the inherently favorable genetic profile of the black population against the development of AMD. Nevertheless, further research regarding potential candidate genes that metformin targets between various ethnic groups is warranted to better account for the different treatment outcomes noted in our study.

### Strength and Limitations

The strengths of this study are that it was the first study to investigate the association between metformin use and either dry or wet AMD. Multiple population-based studies with diverse ethnic backgrounds also enabled subgroup analyses to be performed on ethnicity. Due to the lack of effective treatment for dry AMD at present, the results of this study may prompt further prospective clinical trials exploring the therapeutic effect of metformin on this group of patients.

Several limitations exist in this study. First, previous studies showed disparate risk-lowering effects of metformin at different cumulative and average daily doses. Thus, a dose‒response meta-analysis may elucidate whether the risk of AMD decreases as the metformin dosage increases.^12^^,^^14^^,^^15^^,^^17^ Although stratified analysis according to different dose ranges was demonstrated in four studies,^12^^,^^14^^,^^15^^,^^17^ attempts to synthesize the corresponding data were futile due to the absence of case numbers provided in each stratum of different dosages. Additional studies are therefore required to establish a dose‒response relationship regarding metformin use and the decrease in AMD risk. Second, subgroup analysis dependent on AMD severity could not be carried out due to a lack of studies classifying patients by various stages of AMD. Only one study subcategorized their participants by stages of AMD.^17^ Furthermore, a limited number of studies designated geographic atrophy (GA) as their primary outcome. Jiang et al. performed subgroup analysis according to AMD stage and found that the OR for late AMD was 0.44 (95% CI = 0.58–0.64, *P* = 0.17). However, both GA and neovascular AMD were incorporated in this group, precluding separate analysis of metformin's therapeutic effect. An ongoing phase II randomized clinical trial considering metformin's effect in hindering the progression of GA in nondiabetic patients with AMD is expected to be complete in December 2024, which may further shed light on the treatment benefit of metformin.^47^ Third, the number of studies included in some subgroup analyses was small. For instance, only one study was analyzed for the outcome of wet AMD, which may explain the nonsignificant treatment result of metformin use.^18^ Last, as associations derived from mostly retrospective observational studies do not imply causation, the positive treatment effect should be interpreted cautiously.

## Conclusion

This systematic review and meta-analysis revealed that oral metformin use is associated with decreased odds of AMD in patients with diabetes. Black and Hispanic populations are shown to derive the most benefit from metformin usage. However, no reduction in odds was observed in dry and wet AMD patients taking metformin. Evidence from prospective large-scale clinical trials is needed to confirm this observation.

## Supplementary Material

Supplement 1

## References

[1] Trends in prevalence of blindness and distance and near vision impairment over 30 years: an analysis for the Global Burden of Disease Study. *Lancet Glob Health*. 2021; 9(2): e130–e143. 33275950 10.1016/S2214-109X(20)30425-3 PMC7820390

[2] Wong WL, Su X, Li X, et al. Global prevalence of age-related macular degeneration and disease burden projection for 2020 and 2040: a systematic review and meta-analysis. *Lancet Glob Health*. 2014; 2(2): e106–e116. 25104651 10.1016/S2214-109X(13)70145-1

[3] 3rd, Ferris FL, Fine SL, Hyman L. Age-related macular degeneration and blindness due to neovascular maculopathy. *Arch Ophthalmol*. 1984; 102(11): 1640–1642. 6208888 10.1001/archopht.1984.01040031330019

[4] Campbell JM, Bellman SM, Stephenson MD, Lisy K. Metformin reduces all-cause mortality and diseases of ageing independent of its effect on diabetes control: A systematic review and meta-analysis. *Ageing Res Rev*. 2017; 40: 31–44. 28802803 10.1016/j.arr.2017.08.003

[5] Qu S, Zhang C, Liu D, et al. Metformin Protects ARPE-19 Cells from Glyoxal-Induced Oxidative Stress. *Oxid Med Cell Longev*. 2020; 2020: 1740943. 32695253 10.1155/2020/1740943 PMC7368933

[6] Li Y, Gappy S, Liu X, et al. Metformin suppresses pro-inflammatory cytokines in vitreous of diabetes patients and human retinal vascular endothelium. *PLoS One*. 2022; 17(7): e0268451. 35802672 10.1371/journal.pone.0268451 PMC9269956

[7] Han J, Li Y, Liu X, et al. Metformin suppresses retinal angiogenesis and inflammation in vitro and in vivo. *PLoS One*. 2018; 13(3): e0193031. 29513760 10.1371/journal.pone.0193031 PMC5841739

[8] Zhao X, Liu L, Jiang Y, et al. Protective Effect of Metformin against Hydrogen Peroxide-Induced Oxidative Damage in Human Retinal Pigment Epithelial (RPE) Cells by Enhancing Autophagy through Activation of AMPK Pathway. *Oxid Med Cell Longev*. 2020; 2020: 2524174. 32774666 10.1155/2020/2524174 PMC7397438

[9] Hanus J, Anderson C, Wang S. RPE necroptosis in response to oxidative stress and in AMD. *Ageing Res Rev*. 2015; 24(Pt B): 286–298. 26369358 10.1016/j.arr.2015.09.002 PMC4661094

[10] Eandi CM, Charles Messance H, Augustin S, et al. Subretinal mononuclear phagocytes induce cone segment loss via IL-1β. *Elife*. 2016; 5: e16490. 27438413 10.7554/eLife.16490 PMC4969036

[11] Kaarniranta K, Tokarz P, Koskela A, Paterno J, Blasiak J. Autophagy regulates death of retinal pigment epithelium cells in age-related macular degeneration. *Cell Biology and Toxicol*. 2017; 33(2): 113–128. 10.1007/s10565-016-9371-8 PMC5325845 27900566

[12] Blitzer AL, Ham SA, Colby KA, Skondra D. Association of Metformin Use With Age-Related Macular Degeneration: A Case-Control Study. *JAMA Ophthalmol*. 2021; 139(3): 302–309. 33475696 10.1001/jamaophthalmol.2020.6331 PMC7821082

[13] Brown EE, Ball JD, Chen Z, et al. The Common Antidiabetic Drug Metformin Reduces Odds of Developing Age-Related Macular Degeneration. *Invest Ophthalmol Vis Sci*. 2019; 60(5): 1470–1477. 30973575 10.1167/iovs.18-26422 PMC6736343

[14] Chen YY, Shen YC, Lai YJ, et al. Association between Metformin and a Lower Risk of Age-Related Macular Degeneration in Patients with Type 2 Diabetes. *J Ophthalmol*. 2019; 2019: 1649156. 31781371 10.1155/2019/1649156 PMC6875398

[15] Eton EA, Wubben TJ, Besirli CG, et al. Association of metformin and development of dry age-related macular degeneration in a U.S. insurance claims database. *Eur J Ophthalmol*. 2022; 32(1): 417–423. 33607930 10.1177/1120672121997288 PMC8374004

[16] Gokhale KM, Adderley NJ, Subramanian A, et al. Metformin and risk of age-related macular degeneration in individuals with type 2 diabetes: a retrospective cohort study [published online ahead of print February 3, 2022]. *Br J Ophthalmol*, 10.1136/bjophthalmol-2021-319641. 35115301

[17] Jiang J, Chen Y, Zhang H, et al. Association between metformin use and the risk of age-related macular degeneration in patients with type 2 diabetes: a retrospective study. *BMJ Open*. 2022; 12(4): e054420. 10.1136/bmjopen-2021-054420 PMC9045056 35473747

[18] Lee H, Jeon HL, Park SJ, Shin JY. Effect of Statins, Metformin, Angiotensin-Converting Enzyme Inhibitors, and Angiotensin II Receptor Blockers on Age-Related Macular Degeneration. *Yonsei Med J*. 2019; 60(7): 679–686. 31250582 10.3349/ymj.2019.60.7.679 PMC6597462

[19] Stewart JM, Lamy R, Wu F, Keenan JD. Relationship between Oral Metformin Use and Age-Related Macular Degeneration. *Ophthalmol Retina*. 2020; 4(11): 1118–1119. 32525055 10.1016/j.oret.2020.06.003 PMC7609465

[20] Vergroesen JE, Thee EF, Ahmadizar F, et al. Association of Diabetes Medication With Open-Angle Glaucoma, Age-Related Macular Degeneration, and Cataract in the Rotterdam Study. *JAMA Ophthalmol*. 2022; 140(7): 674–681. 35587864 10.1001/jamaophthalmol.2022.1435 PMC9121302

[21] Romdhoniyyah DF, Harding SP, Cheyne CP, Beare , NAV. Metformin, A Potential Role in Age-Related Macular Degeneration: A Systematic Review and Meta-Analysis. Ophthalmol Ther. 2021; 10(2): 245–260. 33846958 10.1007/s40123-021-00344-3 PMC8079568

[22] Higgins J, Morgan R, Rooney A, et al. Risk Of Bias In Non-randomized Studies - of Exposure (ROBINS-E). Available at: https://www.riskofbias.info/welcome/robins-e-tool. Accessed August 28, 2022.

[23] Higgins JP, Thompson SG. Quantifying heterogeneity in a meta-analysis. *Stat Med*. 2002; 21(11): 1539–1358. 12111919 10.1002/sim.1186

[24] *Trial Sequential Analysis (TSA) [Computer program]* . Version 0.9.5.10 Beta. The Copenhagen Trial Unit, Centre for Clinical Intervention Research, The Capital Region, Copenhagen University Hospital – Rigshospitalet, 2021.

[25] Wetterslev J, Jakobsen JC, Gluud C. Trial Sequential Analysis in systematic reviews with meta-analysis. *BMC Med Res Methodol*. 2017; 17(1): 39. 28264661 10.1186/s12874-017-0315-7 PMC5397700

[26] Kuan V, Warwick A, Hingorani A, et al. Association of Smoking, Alcohol Consumption, Blood Pressure, Body Mass Index, and Glycemic Risk Factors With Age-Related Macular Degeneration: A Mendelian Randomization Study. *JAMA Ophthalmol*. 2021; 139(12): 1299–1306. 34734970 10.1001/jamaophthalmol.2021.4601 PMC8569599

[27] Dekkers OM, Vandenbroucke JP, Cevallos M, et al. COSMOS-E: Guidance on conducting systematic reviews and meta-analyses of observational studies of etiology. *PLoS Med*. 2019; 16(2): e1002742. 30789892 10.1371/journal.pmed.1002742 PMC6383865

[28] Sáenz J, Alba G, Reyes-Quiroz ME, et al. Curcumin enhances LXRα in an AMP-activated protein kinase-dependent manner in human macrophages. *J Nutr Biochem*. 2018; 54: 48–56. 29242172 10.1016/j.jnutbio.2017.11.006

[29] Schulman IG. Liver X receptors link lipid metabolism and inflammation. *FEBS Lett*. 2017; 591(19): 2978–2991. 28555747 10.1002/1873-3468.12702 PMC5638683

[30] Gnanaguru G, Choi AR, Amarnani D, D'Amore PA. Oxidized Lipoprotein Uptake Through the CD36 Receptor Activates the NLRP3 Inflammasome in Human Retinal Pigment Epithelial Cells. *Invest Ophthalmol Vis Sci*. 2016; 57(11): 4704–4712. 27607416 10.1167/iovs.15-18663 PMC5024668

[31] Hoppe G, O'Neil J, Hoff HF, Sears J. Accumulation of oxidized lipid-protein complexes alters phagosome maturation in retinal pigment epithelium. *Cell Mol Life Sci*. 2004; 61(13): 1664–1674. 15224189 10.1007/s00018-004-4080-5 PMC11138549

[32] AnandBabu K, Sen P, Angayarkanni N. Oxidized LDL, homocysteine, homocysteine thiolactone and advanced glycation end products act as pro-oxidant metabolites inducing cytokine release, macrophage infiltration and pro-angiogenic effect in ARPE-19 cells. *PLoS One*. 2019; 14(5): e0216899. 31086404 10.1371/journal.pone.0216899 PMC6516731

[33] Javadzadeh A, Ghorbanihaghjo A, Bahreini E, et al. Serum paraoxonase phenotype distribution in exudative age-related macular degeneration and its relationship to homocysteine and oxidized low-density lipoprotein. *Retina*. 2012; 32(4): 658–666. 22030834 10.1097/IAE.0b013e31822529b1

[34] Strauss O. The retinal pigment epithelium in visual function. *Physiol Rev*. 2005; 85(3): 845–881. 15987797 10.1152/physrev.00021.2004

[35] Hyttinen JM, Petrovski G, Salminen A, Kaarniranta K. 5'-Adenosine monophosphate-activated protein kinase–mammalian target of rapamycin axis as therapeutic target for age-related macular degeneration. *Rejuvenation Res*. 2011; 14(6): 651–660. 22007913 10.1089/rej.2011.1220

[36] Kaarniranta K, Xu H, Kauppinen A. Mechanistical retinal drug targets and challenges. *Adv Drug Deliv Rev*. 2018; 126: 177–184. 29698626 10.1016/j.addr.2018.04.016

[37] Kaarniranta K, Kauppinen A, Blasiak J, Salminen A. Autophagy regulating kinases as potential therapeutic targets for age-related macular degeneration. *Future Med Chem*. 2012; 4(17): 2153–2161. 23190104 10.4155/fmc.12.169

[38] Duncan KG, Bailey KR, Kane JP, Schwartz DM. Human retinal pigment epithelial cells express scavenger receptors BI and BII. *Biochem Biophys Res Commun*. 2002; 292(4): 1017–1022. 11944916 10.1006/bbrc.2002.6756

[39] Duncan KG, Hosseini K, Bailey KR, et al. Expression of reverse cholesterol transport proteins ATP-binding cassette A1 (ABCA1) and scavenger receptor BI (SR-BI) in the retina and retinal pigment epithelium. *Br J Ophthalmol*. 2009; 93(8): 1116–1120. 19304587 10.1136/bjo.2008.144006 PMC3541028

[40] Jr., Brewer HB, Santamarina-Fojo S. New insights into the role of the adenosine triphosphate-binding cassette transporters in high-density lipoprotein metabolism and reverse cholesterol transport. *Am J Cardiol*. 2003; 91(7a): 3e–11e. 10.1016/s0002-9149(02)03382-9 12679197

[41] Van Eck M, Pennings M, Hoekstra M, Out R, Van Berkel TJ. Scavenger receptor BI and ATP-binding cassette transporter A1 in reverse cholesterol transport and atherosclerosis. *Curr Opin Lipidol*. 2005; 16(3): 307–315. 15891392 10.1097/01.mol.0000169351.28019.04

[42] Nieland TJ, Chroni A, Fitzgerald ML, et al. Cross-inhibition of SR-BI- and ABCA1-mediated cholesterol transport by the small molecules BLT-4 and glyburide. *J Lipid Res*. 2004; 45(7): 1256–1265. 15102890 10.1194/jlr.M300358-JLR200

[43] Fisher DE, Klein BE, Wong TY, et al. Incidence of Age-Related Macular Degeneration in a Multi-Ethnic United States Population: The Multi-Ethnic Study of Atherosclerosis. *Ophthalmology*. 2016; 123(6): 1297–1308. 26896123 10.1016/j.ophtha.2015.12.026 PMC4877264

[44] Leske MC, Wu SY, Hennis A, et al. Nine-year incidence of age-related macular degeneration in the Barbados Eye Studies. *Ophthalmology*. 2006; 113(1): 29–35. 16290049 10.1016/j.ophtha.2005.08.012

[45] Weiter JJ, Delori FC, Wing GL, Fitch KA. Relationship of senile macular degeneration to ocular pigmentation. *Am J Ophthalmol*. 1985; 99(2): 185–187. 3970124 10.1016/0002-9394(85)90230-2

[46] Lu F, Liu S, Hao Q, et al. Association Between Complement Factor C2/C3/CFB/CFH Polymorphisms and Age-Related Macular Degeneration: A Meta-Analysis. *Genet Test Mol Biomarkers*. 2018; 22(9): 526–540. 30179527 10.1089/gtmb.2018.0110

[47] ClinicalTrials.gov. Metformin for the Minimization of Geographic Atrophy Progression in Patients with AMD (METforMIN). Updated August 3, 2022. Available at: https://clinicaltrials.gov/ct2/show/NCT02684578. Accessed August 27, 2022.

