# Supplementary material for: Association Between Oral Metformin Use and the Development of Age-Related Macular Degeneration in Diabetic Patients: A Systematic Review and Meta-Analysis
Source: Invest Ophthalmol Vis Sci. 2022 Dec 9;63(13):10. doi: 10.1167/iovs.63.13.10 (PMC9749867; doi:10.1167/iovs.63.13.10)
Supplement: Supplement 1 [file iovs-63-13-10_s001.pdf]

Supplementary material

Supplementary Table 1. PRISMA checklist

Supplementary Table 2. Search Strategies Modified in (a) MEDLINE, (b) Embase, (c) Cochrane Library, and (d) Web of Science

Supplementary Table 3. Adjusted Covariates of Included Studies

Supplementary Table 4. Sensitivity Analysis (a) Leave-one-out Analysis (b) Removing Studies with High Risk of Bias

Supplementary Figure 1. Summary of Risk of Bias

Supplementary Figure 2. Subgroup Analysis of AMD According to AMD subtypes

Supplementary Figure 3. Subgroup Analysis of AMD According to Ethnicity

Supplementary Figure 4. Subgroup Analysis of AMD According to Study Design

Supplementary Figure 5. Subgroup Analysis of AMD According to Sex

Supplementary Figure 6. Subgroup Analysis of AMD According to Ascertainment of AMD

Supplementary Table 1. PRISMA checklist

Section and Topic	Item #	Checklist item	Reported on page No
**TITLE**			
Title	1	Identify the report as a systematic review.	1
**ABSTRACT**			
Abstract	2	See the PRISMA 2020 for Abstracts checklist.	3
**INTRODUCTION**			
Rationale	3	Describe the rationale for the review in the context of existing knowledge.	4
Objectives	4	Provide an explicit statement of the objective(s) or question(s) the review addresses.	4
**METHODS**			
Eligibility criteria	5	Specify the inclusion and exclusion criteria for the review and how studies were grouped for the syntheses.	5-6
Information sources	6	Specify all databases, registers, websites, organisations, reference lists and other sources searched or consulted to identify studies. Specify the date when each source was last searched or consulted.	5
Search strategy	7	Present the full search strategies for all databases, registers and websites, including any filters and limits used.	Supplementary Table 2
Selection process	8	Specify the methods used to decide whether a study met the inclusion criteria of the review, including how many reviewers screened each record and each report retrieved, whether they worked independently, and if applicable, details of automation tools used in the process.	6, Fig. 1
Data collection process	9	Specify the methods used to collect data from reports, including how many reviewers collected data from each report, whether they worked independently, any processes for obtaining or confirming data from study investigators, and if applicable, details of automation tools used in the process.	6
Data items	10a	List and define all outcomes for which data were sought. Specify whether all results that were compatible with each outcome domain in each study were sought (e.g. for all measures, time points, analyses), and if not, the methods used to decide which results to collect.	7
	10b	List and define all other variables for which data were sought (e.g. participant and intervention characteristics, funding sources). Describe any assumptions made about any missing or unclear information.	7
Study risk of bias assessment	11	Specify the methods used to assess risk of bias in the included studies, including details of the tool(s) used, how many reviewers assessed each study and whether they worked independently, and if applicable, details of automation tools used in the process.	6
Effect measures	12	Specify for each outcome the effect measure(s) (e.g. risk ratio, mean difference) used in the synthesis or presentation of results.	7
Synthesis methods	13a	Describe the processes used to decide which studies were eligible for each synthesis (e.g. tabulating the study intervention characteristics and comparing against the planned groups for each synthesis (item #5)).	7
	13b	Describe any methods required to prepare the data for presentation or synthesis, such as handling of missing summary statistics, or data conversions.	7
	13c	Describe any methods used to tabulate or visually display results of individual studies and syntheses.	Table 1
	13d	Describe any methods used to synthesize results and provide a rationale for the choice(s). If meta-analysis was performed, describe the model(s), method(s) to identify the presence and extent of statistical heterogeneity, and software package(s) used.	7
	13e	Describe any methods used to explore possible causes of heterogeneity among study results (e.g. subgroup analysis, meta-regression).	7

Section and Topic	Item #	Checklist item	Reported on page No
	13f	Describe any sensitivity analyses conducted to assess robustness of the synthesized results.	7
Reporting bias assessment	14	Describe any methods used to assess risk of bias due to missing results in a synthesis (arising from reporting biases).	Not applicable
Certainty assessment	15	Describe any methods used to assess certainty (or confidence) in the body of evidence for an outcome.	Nil
**RESULTS**			
Study selection	16a	Describe the results of the search and selection process, from the number of records identified in the search to the number of studies included in the review, ideally using a flow diagram.	8
	16b	Cite studies that might appear to meet the inclusion criteria, but which were excluded, and explain why they were excluded.	Fig. 1
Study characteristics	17	Cite each included study and present its characteristics.	8-9
Risk of bias in studies	18	Present assessments of risk of bias for each included study.	9, Supplementary Fig. 1
Results of individual studies	19	For all outcomes, present, for each study: (a) summary statistics for each group (where appropriate) and (b) an effect estimate and its precision (e.g. confidence/credible interval), ideally using structured tables or plots.	9-10, Fig.2
Results of syntheses	20a	For each synthesis, briefly summarise the characteristics and risk of bias among contributing studies.	9, Supplementary Fig. 1
	20b	Present results of all statistical syntheses conducted. If meta-analysis was done, present for each the summary estimate and its precision (e.g. confidence/credible interval) and measures of statistical heterogeneity. If comparing groups, describe the direction of the effect.	9-10, Fig. 2, Supplementary Fig. 2-6
	20c	Present results of all investigations of possible causes of heterogeneity among study results.	9-10, Table 2
	20d	Present results of all sensitivity analyses conducted to assess the robustness of the synthesized results.	10, Supplementary table 4
Reporting biases	21	Present assessments of risk of bias due to missing results (arising from reporting biases) for each synthesis assessed.	Not applicable
Certainty of evidence	22	Present assessments of certainty (or confidence) in the body of evidence for each outcome assessed.	Nil
**DISCUSSION**			
Discussion	23a	Provide a general interpretation of the results in the context of other evidence.	11-12
	23b	Discuss any limitations of the evidence included in the review.	14-15
	23c	Discuss any limitations of the review processes used.	14-15
	23d	Discuss implications of the results for practice, policy, and future research.	15

Section and Topic	Item #	Checklist item	Reported on page No
**OTHER INFORMATION**			
Registration and protocol	24a	Provide registration information for the review, including register name and registration number, or state that the review was not registered.	5
	24b	Indicate where the review protocol can be accessed, or state that a protocol was not prepared.	5
	24c	Describe and explain any amendments to information provided at registration or in the protocol.	Nil
Support	25	Describe sources of financial or non-financial support for the review, and the role of the funders or sponsors in the review.	2
Competing interests	26	Declare any competing interests of review authors.	2
Availability of data, code and other materials	27	Report which of the following are publicly available and where they can be found: template data collection forms; data extracted from included studies; data used for all analyses; analytic code; any other materials used in the review.	15

Supplementary Table 2

Search Strategies Modified in Ovid MEDLINE (a), Ovid Embase (b), Cochrane Library (c), and Web of Science (d)

a. Search strategy in MEDLINE (via Ovid MEDLINE(R), 1946 to present; search date: 2022/08/03)

#	Search syntax	Citations found
1	((macul* or retina* or choroid*) adj4 degenera*).tw.	35321
2	((macul* or retina* or choroid*) adj4 neovascular*).tw.	14351
3	((macul* or geograph*) adj4 atroph*).tw.	2890
4	maculopath*.tw.	5156
5	(AMD or ARMD or CNV or GA).tw.	75602
6	exp Macular Degeneration/	28615
7	exp Retinal Degeneration/	48854
8	exp Choroidal Neovascularization/	6547
9	exp Geographic Atrophy/	960
10	exp Retinal Neovascularization/	3326
11	1 or 2 or 3 or 4 or 5 or 6 or 7 or 8 or 9 or 10	134451
12	metformin*.tw.	25211
13	exp Metformin/	16711
14	12 or 13	27192
15	11 and 14	109

b. Search strategy in Embase (via Ovid Embase, 1974 to present; search date: 2022/08/03)

#	Search syntax	Citations found
1	((macul* or retina* or choroid*) adj4 degenera*).tw.	46695
2	((macul* or retina* or choroid*) adj4 neovascular*).tw.	18689
3	(geograph* adj4 atroph*).tw.	2471
4	exp retina degeneration/	49920
5	exp retina neovascularization/	6766
6	exp retina maculopathy/	56365
7	exp macular degeneration/	22573
8	exp retina macula age related degeneration/	13000
9	exp retina macula degeneration/	22573
10	exp geographic atrophy/	2294

11	maculopath*.tw.	6525
12	1 or 2 or 3 or 4 or 5 or 6 or 7 or 8 or 9 or 10 or 11	114040
13	metformin*.tw.	42701
14	exp metformin/	77142
15	13 or 14	80115
16	12 and 15	299

c. Search strategy in Cochrane Library (via Cochrane Collaboration; search date: 2022/08/03)

#	Search syntax	Citations found
1	MeSH descriptor: [Macular Degeneration] explode all trees	2803
2	MeSH descriptor: [Geographic Atrophy] explode all trees	155
3	MeSH descriptor: [Retinal Degeneration] explode all trees	2947
4	MeSH descriptor: [Retinal Neovascularization] explode all trees	86
5	MeSH descriptor: [Choroidal Neovascularization] explode all trees	452
6	(macula* or retina* or choroid*) near/4 degenerat*	3829
7	(macula* or retina* or choroid*) near/4 neovascul*	2434
8	geograph* near/4 atroph*	449
9	maculopath*	476
10	AMD or ARMD or CNV or GA	24081
11	#1 or #2 or #3 or #4 or #5 or #6 or #7 or #8 or #9 or #10	2393
12	Metformin*	12116
13	MeSH descriptor: [Metformin] explode all trees	4517
14	#12 or #13	12116
15	#11 and #14	123

d. Search strategy in Web of Science Core Collection (2015 to present; search date: 2022/08/03)

#	Search syntax	Citations found
1	(((((TS=((macula* or retina* or choroid*) near/4 degenerat*)) OR TS=((macula* or retina* or choroid*) near/4 neovascul*)) OR TS=(maculopath*)) OR TS=(geograph* near/4 atroph*)) OR TS=(AMD or ARMD or CNV or GA)) AND TS=(metformin*)	102

Supplementary Table 3. Adjusted covariates of included studies

Source (Country)	Age	Sex	BMI	Race	Socioeconomic data	Smoking	CAD	Anemia	DM	DR	HTN	Dyslipidemia	CCI	Other variables
Brown et al,[13] 2019 (US)	•							•			•		•	
Chen et al,[14] 2019 (Taiwan)	•	•					•			•	•	•		Obesity, CKD, poor DM control (insulin use), antidiabetics, antihypertensives, statin
Lee et al,[18] 2019 (South Korea)					•		•		•		•	•	•	Liver disease, PVD, medication history, CVA
Steward et al,[19] 2020 (US)	•	•		•	•	•				•				Metabolic syndrome (DR)
Blitzer et al,[12] 2021 (US)	•	•				•			•	•	•	•	•	Statin, DR (PDR, NPDR), obesity, region
Eton et al,[15] 2022 (US)	•	•		•	•			•		•	•	•		Region, NPDR, stroke, PVD, CKD, liver/lung disease, malignancy, DCSI
Gokhale et al,[16] 2022 (UK)	•	•	•	•	•	•							•	SBP, DM complications, HbA1c, statin, CVD, CKD, hypothyroidism
Jiang et al,[17] 2022 (China)	•	•	•			•			•		•	•		FBG, HbA1c, UA, Cr
Vergroesen et al,[20] 2022 (Netherlands)	•	•	•			•			•		•	•	•	Statin, antihypertensives

BMI, body mass index; CAD, coronary artery disease; CCI, Charlson Comorbidity Index; CKD, chronic kidney disease; Cr, creatinine; CVA, cardiovascular accident; CVD, cardiovascular disease; DCSI, Diabetes Complications Severity Index; DM, diabetes mellitus; DR, diabetic retinopathy; FBG, fasting blood glucose; HTN, hypertension; NPDR, nonproliferative diabetic retinopathy; PDR, proliferative diabetic retinopathy; PVD, peripheral vascular disease; SBP, systolic blood pressure; UA, uric acid; UK, United Kingdom; US, United States.

Supplementary Table 4. Sensitivity Analysis

(a) Leave-one out Analysis

Study removed	Age-related macular degeneration		
	**Pooled OR (95% CI)**	**p-value**	**I^2 (%)**
Blitzer et al.	0.73 (0.57, 0.95)	0.02*	96
Brown et al.	0.82 (0.70, 0.95)	<0.01**	96
Chen et al.	0.91 (0.82, 1.01)	0.09	90
Eton et al.	0.73 (0.59, 0.91)	<0.01**	95
Gokhale et al.	0.77 (0.66, 0.91)	<0.01**	96
Jiang et al.	0.85 (0.74, 0.98)	0.03*	96
Lee et al.	0.77 (0.66, 0.90)	<0.01**	96
Stewart et al.	0.82 (0.71, 0.96)	0.01*	96
Vergroesen et al.	0.82 (0.71, 0.95)	<0.01**	96

*$p < 0.05$; **$p < 0.01$

(b) Removing Studies with High Risk of Bias

Studies removed	Remaining no. of studies	Age-related macular degeneration		
		**Pooled OR (95% CI)**	***p*-value**	**I^2 (%)**
Brown et al., Chen et al., Eton et al,. Lee et al.	5	0.78 (0.64, 0.95)	0.01*	87

*$p < 0.05$; **$p < 0.01$

Supplementary Figure 1. Summary of Risk of Bias (ROBINS-E)

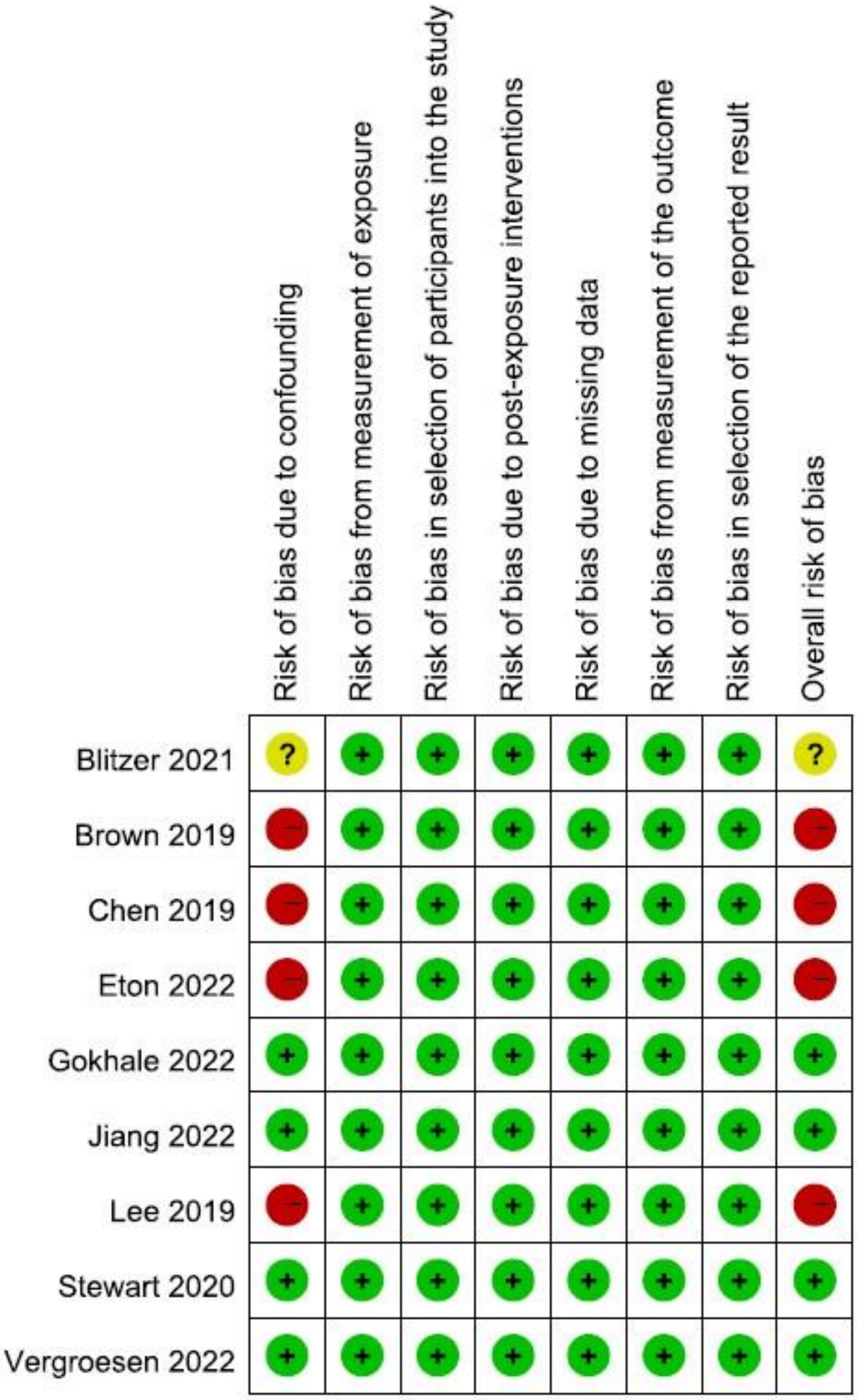

The green plus circle denotes low risk of bias, question mark circle denotes some concern, and red minus circle high risk of bias.

Supplementary Figure 2. Subgroup Analysis of AMD According to AMD subtypes

Study or Subgroup	log[Odds Ratio]	SE	Weight	Odds Ratio IV, Random, 95% CI
**1.2.1 Dry**				
Eton 2022	0.077	0.0193	38.0%	1.08 [1.04, 1.12]
Lee 2019	0.4824	0.236	27.7%	1.62 [1.02, 2.57]
Stewart 2020	-0.5276	0.127	34.3%	0.59 [0.46, 0.76]
**Subtotal (95% CI)**			**100.0%**	**0.98 [0.62, 1.57]**
Heterogeneity: Tau² = 0.15; Chi² = 25.28, df = 2 (P < 0.00001); I² = 92%				
Test for overall effect: Z = 0.08 (P = 0.94)				
**1.2.2 Wet**				
Lee 2019	0.0296	0.1289	100.0%	1.03 [0.80, 1.33]
**Subtotal (95% CI)**			**100.0%**	**1.03 [0.80, 1.33]**
Heterogeneity: Not applicable				
Test for overall effect: Z = 0.23 (P = 0.82)				

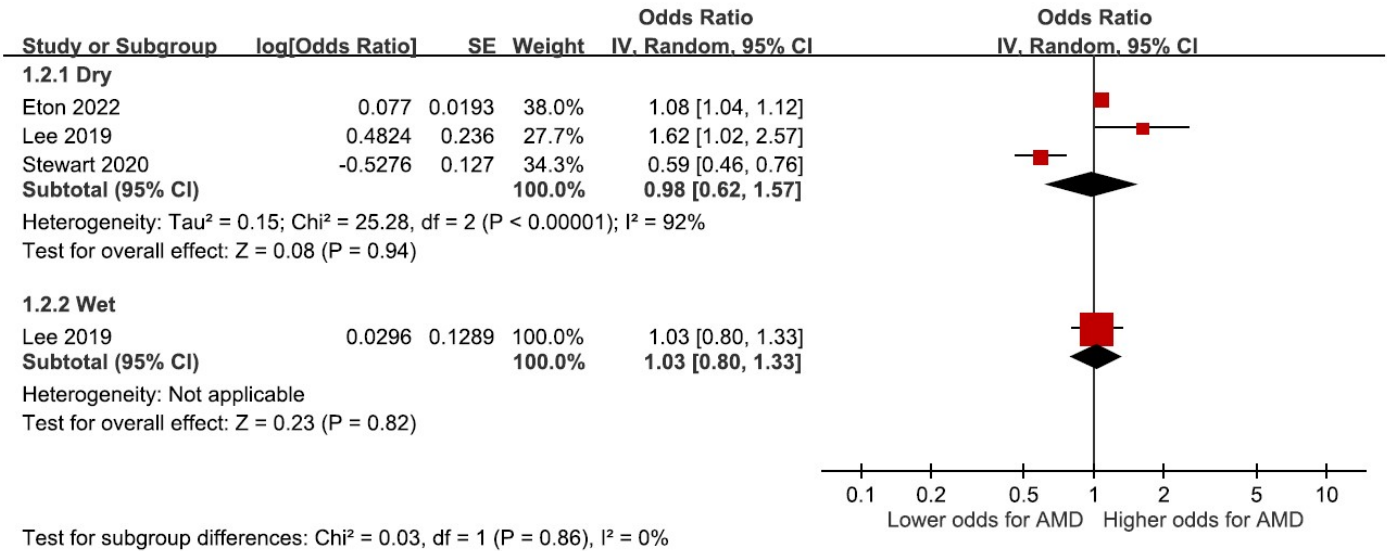

Test for subgroup differences: Chi² = 0.03, df = 1 (P = 0.86), I² = 0%

AMD, age-related macular degeneration; CI, confidence interval; IV, inverse variance; SE, standard error.

Supplementary Figure 3. Subgroup Analysis of AMD According to Ethnicity

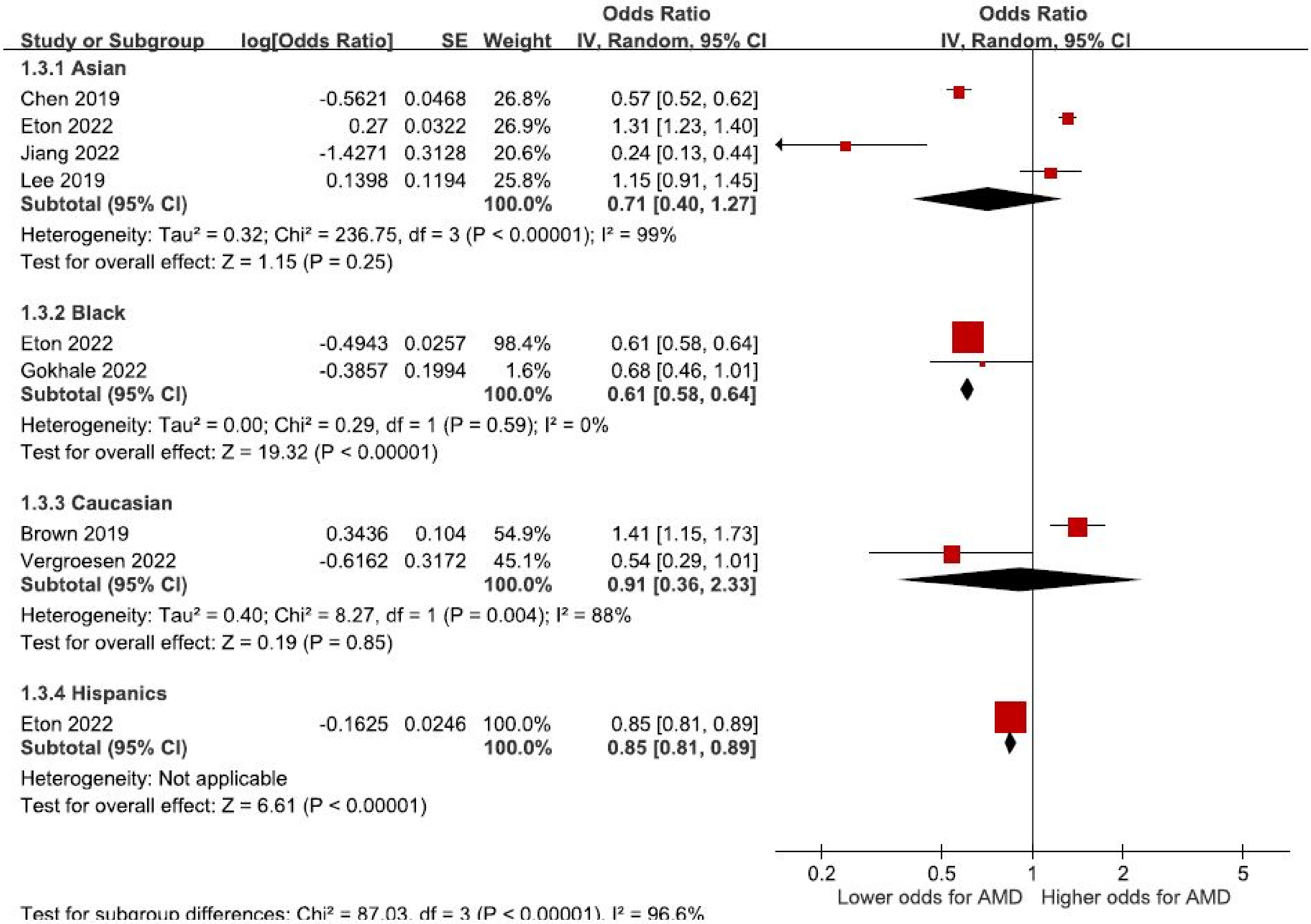

AMD, age-related macular degeneration; CI, confidence interval; IV, inverse variance; SE, standard error.

Supplementary Figure 4. Subgroup Analysis of AMD According to Study Design

Study or Subgroup	log[Odds Ratio]	SE	Weight	Odds Ratio IV, Random, 95% CI
**1.4.1 Cohort studies**				
Chen 2019	-0.5621	0.0468	24.0%	0.57 [0.52, 0.62]
Eton 2022	0.077	0.0193	24.3%	1.08 [1.04, 1.12]
Gokhale 2022	0.01	0.0532	23.9%	1.01 [0.91, 1.12]
Jiang 2022	-1.4271	0.3128	14.0%	0.24 [0.13, 0.44]
Vergroesen 2022	-0.6162	0.3172	13.8%	0.54 [0.29, 1.01]
**Subtotal (95% CI)**			**100.0%**	**0.67 [0.47, 0.95]**
Heterogeneity: Tau² = 0.13; Chi² = 183.52, df = 4 (P < 0.00001); I² = 98%				
Test for overall effect: Z = 2.23 (P = 0.03)				
**1.4.2 Others**				
Blitzer 2021	-0.0513	0.0109	36.7%	0.95 [0.93, 0.97]
Brown 2019	-0.3567	0.182	16.2%	0.70 [0.49, 1.00]
Lee 2019	0.1398	0.1194	23.8%	1.15 [0.91, 1.45]
Stewart 2020	-0.3567	0.123	23.3%	0.70 [0.55, 0.89]
**Subtotal (95% CI)**			**100.0%**	**0.88 [0.73, 1.07]**
Heterogeneity: Tau² = 0.03; Chi² = 11.51, df = 3 (P = 0.009); I² = 74%				
Test for overall effect: Z = 1.29 (P = 0.20)				

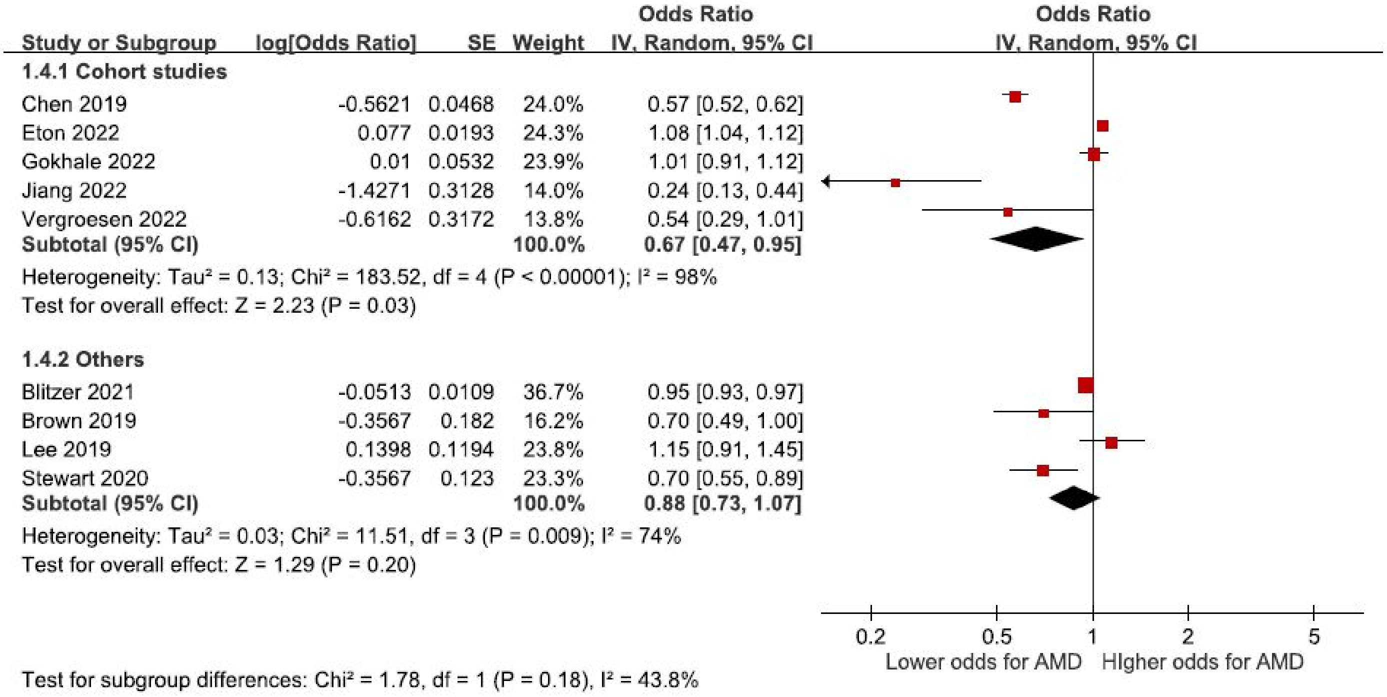

Test for subgroup differences: Chi² = 1.78, df = 1 (P = 0.18), I² = 43.8%

AMD, age-related macular degeneration; CI, confidence interval; IV, inverse variance; SE, standard error.

Supplementary Figure 5. Subgroup Analysis of AMD According to Sex

Study or Subgroup	log[Odds Ratio]	SE	Weight	Odds Ratio IV, Random, 95% CI
**1.5.1 Male**				
Brown 2019	-0.3425	0.061	23.5%	0.71 [0.63, 0.80]
Chen 2019	0.0296	0.0359	26.7%	1.03 [0.96, 1.11]
Eton 2022	-0.1393	0.0119	28.5%	0.87 [0.85, 0.89]
Gokhale 2022	0.0677	0.0771	21.2%	1.07 [0.92, 1.24]
**Subtotal (95% CI)**			**100.0%**	**0.91 [0.79, 1.04]**
Heterogeneity: Tau² = 0.02; Chi² = 38.94, df = 3 (P < 0.00001); I² = 92%				
Test for overall effect: Z = 1.42 (P = 0.16)				
**1.5.2 Female**				
Blitzer 2021	0.1133	0.0139	61.9%	1.12 [1.09, 1.15]
Gokhale 2022	-0.0202	0.0666	38.1%	0.98 [0.86, 1.12]
**Subtotal (95% CI)**			**100.0%**	**1.06 [0.94, 1.21]**
Heterogeneity: Tau² = 0.01; Chi² = 3.85, df = 1 (P = 0.05); I² = 74%				
Test for overall effect: Z = 0.96 (P = 0.34)				

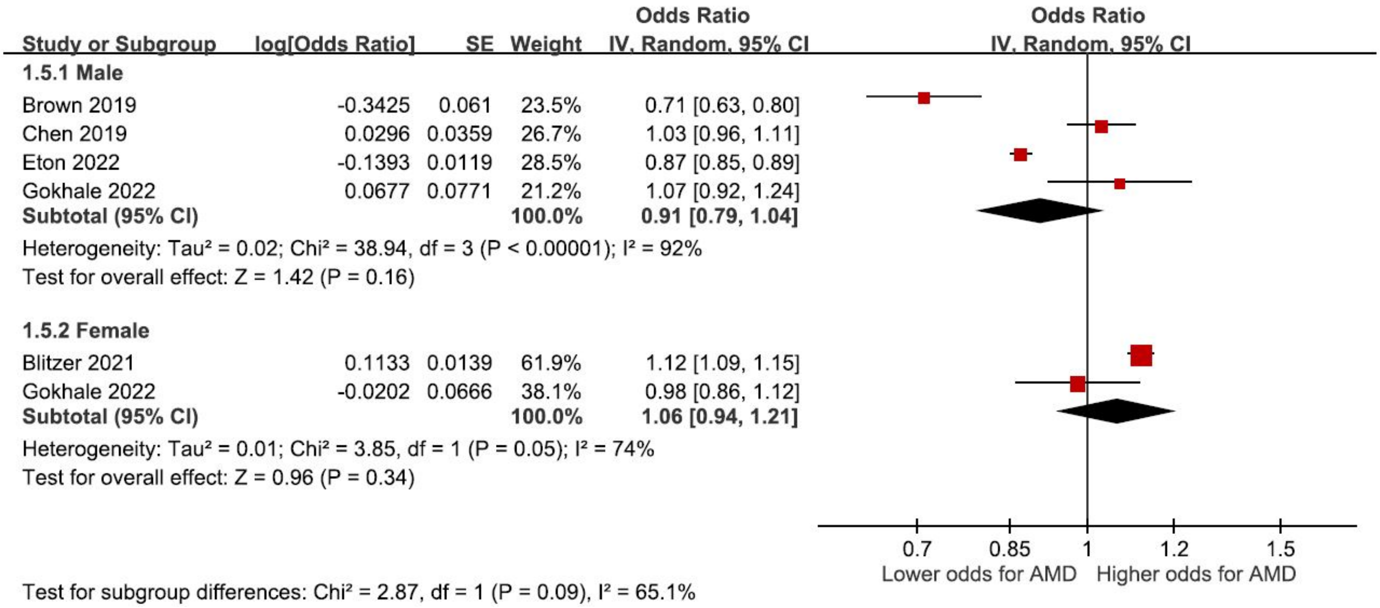

Test for subgroup differences: Chi² = 2.87, df = 1 (P = 0.09), I² = 65.1%

AMD, age-related macular degeneration; CI, confidence interval; IV, inverse variance; SE, standard error.

Supplementary Figure 6. Subgroup Analysis of AMD According to Ascertainment of AMD

Study or Subgroup	log[Odds Ratio]	SE	Weight	Odds Ratio IV, Random, 95% CI
**1.6.1 ICD codes**				
Blitzer 2021	-0.0513	0.0109	19.3%	0.95 [0.93, 0.97]
Brown 2019	-0.3567	0.182	10.2%	0.70 [0.49, 1.00]
Chen 2019	-0.5621	0.0468	18.3%	0.57 [0.52, 0.62]
Eton 2022	0.077	0.0193	19.2%	1.08 [1.04, 1.12]
Jiang 2022	-1.4271	0.3128	5.3%	0.24 [0.13, 0.44]
Lee 2019	0.1398	0.1194	14.0%	1.15 [0.91, 1.45]
Stewart 2020	-0.3567	0.123	13.7%	0.70 [0.55, 0.89]
**Subtotal (95% CI)**			**100.0%**	**0.79 [0.67, 0.93]**
Heterogeneity: Tau² = 0.04; Chi² = 193.64, df = 6 (P < 0.00001); I² = 97%				
Test for overall effect: Z = 2.83 (P = 0.005)				
**1.6.2 Clinical diagnosis**				
Vergroesen 2022	-0.6162	0.3172	100.0%	0.54 [0.29, 1.01]
**Subtotal (95% CI)**			**100.0%**	**0.54 [0.29, 1.01]**
Heterogeneity: Not applicable				
Test for overall effect: Z = 1.94 (P = 0.05)				

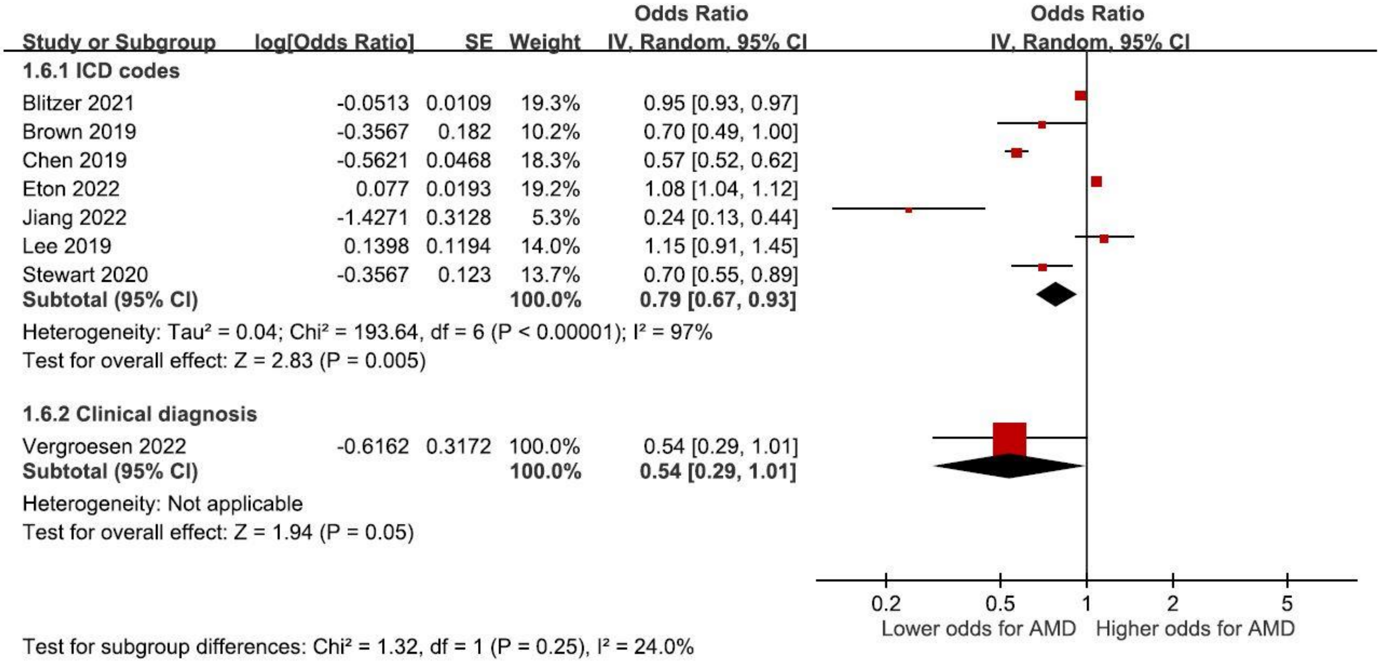

Test for subgroup differences: Chi² = 1.32, df = 1 (P = 0.25), I² = 24.0%

AMD, age-related macular degeneration; CI, confidence interval; IV, inverse variance; SE, standard error.
